# Client involvement in a web-based intervention targeting substance abuse treatment in the criminal justice system

**DOI:** 10.1186/1940-0640-10-S1-A61

**Published:** 2015-02-20

**Authors:** Stephanie Spohr, Mayra Rodriguez, Jennifer Lerch, Faye S Taxman, Scott T Walters

**Affiliations:** 1 Behavioral and Community Health, University of North Texas Health Science Center, Fort Worth, TX, 76107, USA; 2 Criminology, Law & Society, George Mason University, Fairfax, VA, 22030, USA

## Background

Although substance use is common among people in the U.S. criminal justice system, only a minority of probationers actually initiate treatment.

## Methods

This presentation discusses preliminary data from a randomized controlled trial that is testing the use of a two-session, web-based intervention and an in-person counselor-driven intervention to increase motivation for substance abuse treatment among people on probation.

## Results

This study uses data from the first 84 clients who completed the web-based program. The data provided from this study covers probationer involvement with the MAPIT intervention in relation to early goal planning and electronic reminder selection on positive 2-month outcomes. In terms of early goal planning, clients were most likely to select goals such as “Make a list of things I could do to stay sober” (62%); “Write down the date and time of my first PO visit” (52%); and “Get a binder to keep my probation documents in” (50%). In terms of reminder preference, 51 percent wanted to receive text or email reminders about their goals (31% requested text; 20% requested email). Most clients wanted reminders early in the week, and in the morning. Women, low/moderate risk, and older clients (> 35 years old) were more likely to ask for reminders. Those who selected to receive email reminders selected the greatest number of goals (M = 5.3) compared to those who chose text (M = 4.1) or no reminder (M = 3.0), F = 6.20, p = .004. In term of positive 2-month outcomes, probationers in the email reminder group were significantly more likely to be abstinent and were significantly more likely to initiate treatment at follow-up (χ^2^ = 6.25, df = 2, p < .05 and χ^2^ = 6.51, df = 2, p < .05, respectively), compared to the text reminder and no reminder groups. Early signs of non-responding were indicated by participants choosing the minimum number of goals (2), not selecting to receive electronic reminders, and not completing any goals by visit two, and were indicative of poorer outcomes.

## Conclusion

Probation offenders have a variety of reasons for wanting to finish probation and complete treatment. Clients are able to identify specific goals for the next month, and most clients want to be reminded about those goals. This information can assist probation agents in helping clients to identify ways to be more deliberate in achieving early probation goals.

## Trial registration

NCT01891656

